# Development and characterization of a functional oat milk-based cereal dessert fortified with microencapsulated *Lactobacillus plantarum* and Balangu Shirazi (*Lallemantia royleana*) gum

**DOI:** 10.1007/s10068-026-02302-w

**Published:** 2026-09-18

**Authors:** Milad Daneshniya, Parham Joolaei Ahranjani, Kamine Dehghan, Mohammad Hossein Maleki, Farzaneh Abdolmaleki, Giovanna Ferrentino

**Affiliations:** 1 Department of Food Science and Technology, Qa.C., Islamic Azad University, Qazvin, Iran; 2 Faculty of Agricultural, Environmental and Food Sciences, Free University of Bolzano, Piazza Università, 1, 39100 Bolzano, Italy; 3 Department of Food Science and Technology, SR.C., Islamic Azad University, Tehran, Iran

**Keywords:** Functional food, Oat milk, Hydrocolloids, Balangu shirazi, Probiotic, Antioxidant activity

## Abstract

This study aimed to develop a functional oat milk–based cereal dessert fortified with microencapsulated *Lactiplantibacillus plantarum* and Balangu Shirazi (*Lallemantia royleana*) gum (BSG), and to evaluate its physicochemical, microbiological, sensory, and textural stability during refrigerated storage. BSG was characterized as a polysaccharide-rich hydrocolloid (moisture 6.23%, ash 7.12%, protein 2.08%, sugars 64.71%) with notable phenolic content (4.49%) and antioxidant activity (21.01% DPPH inhibition). DLS analysis of the diluted dispersion revealed a dominant colloidal population with a hydrodynamic diameter of 46.08 nm and a polydispersity index of 0.12 ± 0.02. This measurement reflected the colloidal species present in the dispersion rather than the dimensions of intact probiotic-containing microcapsules. Over 21 days at 4 °C, the combined formulations showed improved moisture retention and compositional stability, maintaining high presumptive LAB counts during storage. Within the probiotic-containing formulations, increasing BSG concentration was associated with greater moisture retention and higher ash and protein contents. Principal component analysis indicated that the combined formulations mitigated storage-induced sensory deterioration, while textural properties shifted toward softer yet acceptable structures. Overall, the combined incorporation of BSG and microencapsulated *L. plantarum* enabled the development of a stable dairy-free cereal dessert with improved storage performance. While this study focused on evaluating the combined effect of both treatments, future work isolating BSG-only and probiotic-only formulations could offer further insight into their individual contributions to product stability.

**Supplementary Information:**

The online version contains supplementary material available at https://doi.org/10.1007/s10068-026-02302-w.

## Introduction

The demand for plant-based functional foods has increased substantially in recent years, as consumers seek alternatives to dairy products that address lactose intolerance, dietary restrictions, and a preference for sustainable food systems (Daneshniya et al., 2023). Oats are widely used in these formulations because they are naturally lactose-free and rich in β-glucan, a soluble fiber associated with improved serum cholesterol levels and reduced cardiovascular disease risk (EFSA, 2011). Their mild flavor, nutritional value, and high water-binding capacity make oat-based matrices suitable for developing beverages, semi-solid foods, and desserts enriched with health-promoting components (Chen et al., 2020).

Probiotics, defined as live microorganisms that, when administered in adequate amounts, confer a health benefit on the host (Reid et al., 2019), remain one of the most established categories of functional foods. *Lactobacillus plantarum* (*Lactiplantibacillus plantarum*) is of particular interest due to its tolerance to acidic environments, ability to colonize the gastrointestinal tract, and demonstrated antioxidant and antimicrobial activities (Aziz et al., 2023a, b). However, one of the major challenges in formulating probiotic foods is ensuring adequate cell viability during processing, storage, and gastrointestinal transit. Microencapsulation has been shown to effectively protect probiotic cells from harsh environmental conditions, thereby enhancing their survival and stability (Brinques et al., 2011).

Balangu Shirazi (*Lallemantia royleana*) seed has recently attracted attention as a natural plant-derived hydrocolloid with promising functional and bioactive properties for use in food matrices or their packaging (Zandi et al., 2025). Its seed gum contains high-molecular-weight polysaccharides with thickening, stabilizing, and surface-active characteristics suitable for improving texture and structure in food matrices (Farhadi, 2017; Sardarodiyan et al., 2020). Additionally, the gum exhibits antioxidant and antimicrobial properties (Sardarodiyan et al., 2019), suggesting potential synergistic effects when combined with probiotic cultures or used in the development of functional foods. In addition, *Lactiplantibacillus plantarum* was selected as the target strain based on its well-documented tolerance to acidic environments, its established probiotic, antioxidant, and antimicrobial activities, and its demonstrated efficacy in oat- and cereal-based food matrices, as reported in previous studies (Aziz et al., 2023a, b; Meena et al., 2025). Within the present formulation, this strain was intended to confer probiotic functionality to the final product. In contrast, microencapsulation was employed as a protective strategy to enhance cell viability and structural integrity throughout processing and subsequent refrigerated storage.

Despite the growing interest in plant-based probiotic foods, few studies have explored the development of a semi-solid oat milk–based cereal dessert system incorporating microencapsulated *Lactiplantibacillus plantarum* and Balangu Shirazi gum (BSG).

Existing studies have examined fermented oat beverages or microencapsulated probiotics separately. However, the use of *L. royleana* gum as both a structuring agent and a functional ingredient in a cereal-based dessert remains underexplored (Marchwińska et al., 2023; Szydłowska et al., 2021). Addressing this gap is important because combining these components may enhance probiotic viability, improve product texture, and contribute additional functional properties such as antioxidant activity. The primary objective of the study was to develop and characterize a functional oat-based dessert that combines microencapsulated *L. plantarum* with BSG, and to investigate the effect of increasing BSG concentration in a formulation with a constant probiotic load. Therefore, the present study aims to formulate an oat-based cereal dessert containing microencapsulated *L. plantarum* and *L. royleana* gum and to evaluate its physicochemical characteristics, probiotic survival, and functional potential. This work contributes to the development of next-generation plant-based functional desserts and expands the current scientific understanding of BSG in probiotic food systems.

## Materials and methods

### Chemicals and microorganism

Balangu Shirazi (*Lallemantia royleana*) seeds were obtained from a local supplier in Gorgan, Iran. Seeds were stored under dry conditions (25 °C, relative humidity <50%) until use. *Lactobacillus plantarum* (ATCC 1058) was obtained from the Persian Type Culture Collection (PTCC, Tehran, Iran). Sodium alginate (viscosity 200–400 mPa·s, medium molecular weight), calcium chloride dihydrate, DPPH (≥ 95% purity), de Man, Rogosa and Sharpe (MRS) broth and agar, yeast glucose chloramphenicol (YGC) agar, diethyl ether, petroleum ether (boiling range 40–60 °C), sulfuric acid (98%), sodium hydroxide, boric acid, copper sulfate, sodium sulfate, phenolphthalein, and all other analytical reagents were purchased from Merck (Germany). All experiments were performed in three technical replicates, and the results were reported as the mean of the replicates ± standard deviation (SD).

### Preparation of Balangu Shirazi (*Lallemantia royleana*) seed gum

Gum extraction was conducted following a modified hot-water extraction method (Abdolmaleki et al., 2023). Cleaned seeds were dispersed in distilled water at a seed-to-water ratio of 1:59 (w/v) and hydrated at 85 °C under continuous stirring (500 rpm) using a magnetic stirrer (IKA, RCT basic, Germany) for 20 min. The pH of the dispersion was monitored using a calibrated digital pH meter (METTLER TOLEDO, FiveGo, USA) and maintained at 7.0 ± 0.1 without chemical adjustment. The selected extraction temperature and hydration time were based on preliminary optimization to maximize mucilage yield while minimizing thermal degradation of polysaccharides. The hydrated suspension was centrifuged at 10,000 × g for 15 min at 4 °C using a refrigerated centrifuge (Labtron, LRF-F10, UK). The supernatant containing soluble polysaccharides was carefully collected and filtered through a 20 µm mesh (Fisher Scientific, UK). The filtrate was dried in a forced-air oven (Esco, Singapore) at 60 °C until a constant weight was reached. Moisture content of the dried gum was confirmed to be below 10%. The dried gum sheets were milled using a laboratory grinder (Pars, Omega, Iran), passed through a 250 µm sieve (Fisher Scientific, UK), vacuum-packed, and stored in a desiccator at ambient temperature until further use.

### Microbial suspension

Frozen stock cultures of *Lactobacillus plantarum* (ATCC 1058) stored at −80 °C in MRS broth supplemented with 20% (v/v) glycerol were reactivated by two successive transfers in sterile MRS broth. Each activation step was performed by inoculating 1% (v/v) culture into 100 mL MRS broth in 250 mL Erlenmeyer flasks and incubating at 37 °C for 18 h under anaerobic conditions using an anaerobic jar system. Bacterial growth was monitored spectrophotometrically at 600 nm using a UV–Vis spectrophotometer (BioBase, BK-D560, China). The late logarithmic phase was determined from preliminary growth curve analysis (OD600 vs time). Cells were harvested at an OD600 corresponding to approximately 10⁹ CFU/mL based on the previously established OD600–CFU calibration curve. Cultures were centrifuged at 5,000 × g for 10 min at 4 °C using a refrigerated centrifuge. The pellet was washed twice with sterile 0.9% (w/v) saline solution to remove residual growth medium and resuspended in sterile saline. Cell concentration was adjusted to approximately 10⁹ CFU/mL using the OD600 calibration curve and was confirmed by plate counting on MRS agar after incubation at 37 °C for 48–72 h under anaerobic conditions. The growth phase was strictly controlled as encapsulation efficiency and stress tolerance are strongly influenced by the bacterial physiological state (Portilha-Cunha et al., 2020).

### Microencapsulation of probiotic bacteria

Microencapsulation was performed using an emulsion–external ionic gelation technique (Sheu & Marshall, 1993; Chan, 2011). A 4% (w/v) sodium alginate solution was prepared in distilled water under continuous stirring (500 rpm) using a magnetic stirrer and sterilized by autoclaving (121 °C, 15 min). The solution was degassed prior to use to prevent air bubbles from forming. 20 mL of standardized probiotic suspension (≈10⁹ CFU/mL) was aseptically mixed with 100 mL sterile alginate solution. The mixture was introduced dropwise using a sterile syringe fitted with a 21-gauge needle (Hakimsyringe, Iran) into 500 mL of canola oil containing 1% (w/v) Tween 80, under continuous stirring at 200 rpm with a magnetic stirrer. Emulsification was maintained for 20 min to achieve uniform dispersion of aqueous droplets. A cold 0.1 M CaCl_2_ solution (4 °C) was then added slowly under continuous stirring to initiate ionic crosslinking between Ca^2^⁺ and the guluronic acid residues of alginate. The system was maintained under agitation for an additional 15 min to ensure complete gelation. Microcapsules were separated by centrifugation at 2000 × g for 5 min at 4 °C using a refrigerated centrifuge. The pellet was washed twice with sterile 0.9% saline to remove residual oil and excess calcium ions and stored at 4 °C until further use. To determine encapsulation efficiency, 1 g of freshly prepared microcapsules was dispersed in 9 mL of sterile 0.2 M sodium citrate buffer (pH 6.8) and vortexed for 10 min using a vortex mixer (Lachoi, China) to dissolve the alginate matrix and release entrapped cells.

The suspension was serially diluted in sterile saline and plated on MRS agar. Plates were incubated at 37 °C for 72 h under anaerobic conditions. Colonies were counted and expressed as CFU/g. The encapsulation efficiency was determined based on the following equation:

1 $$ EE\% = \frac{{N_{encapsulated} }}{{N_{initial} }} $$

where *N*_*encapsulated*_ is the viable cells released from capsules, and *N*_*initial*_ is the initial viable cell count.

### Oat milk-based cereal dessert preparation

Oat groats were soaked in distilled water at a 1:3 (w/v) ratio for 2 h at ambient temperature (25 ± 2 °C) to facilitate hydration and softening. The soaked grains were blended for 30 s using a laboratory blender to obtain a homogeneous slurry. The slurry was filtered through sterile muslin cloth to extract oat milk. The dessert formulation consisted of 200 g oat milk, 15 g corn starch, 10 g sucrose (5% w/w), and 0.3% (w/w) gelatin. All ingredients were mixed under continuous stirring using a hot-plate magnetic stirrer. The mixture was heated to 85 °C and maintained at this temperature for 15 min, with constant agitation to ensure complete starch gelatinization and reduce the microbial load. Temperature was monitored using a thermometer. After heat treatment, the mixture was cooled to 40 °C under gentle stirring at ambient conditions. Balangu Shirazi gum (BSG) was incorporated at concentrations of 0.60, 1.25, or 2.50 g, depending on the treatment group. When the temperature decreased to 30 °C, 1 g of freshly prepared microcapsules (≈10⁹ CFU/g) was aseptically added and gently mixed to prevent capsule rupture. The final formulations were poured into sterile containers and stored at 4 °C in a refrigeration unit for 21 days, and were as follows: F_0_ was the control (oat-based cereal dessert prepared with oat milk, without microencapsulated *L. plantarum* and BSG, F_1_ contained microencapsulated *L. plantarum* and 0.60 g BSG, F_2_ contained *L. plantarum* and 1.25 g BSG, and F_3_ contained *L. plantarum* and 2.50 g BSG. All formulations were prepared in triplicate, independent batches (Homayoonfal et al., 2015).

### Hydrodynamic diameter and polydispersity index of the dispersed colloidal fraction

The hydrodynamic diameter and polydispersity index (PDI) of the colloidal species detected in the diluted dispersion were determined by dynamic light scattering (DLS) using a Zetasizer instrument (Malvern Instruments, Worcestershire, UK) equipped with a 633 nm He–Ne laser and a backscattering detection angle of 173°. Samples were diluted 1:50 with deionized water to minimize multiple scattering effects and equilibrated at 25 °C for 2 min prior to analysis. Measurements were performed in triplicate, with each replicate consisting of three consecutive runs. Particle size distribution was evaluated on a volume-weighted basis, and the hydrodynamic diameter was reported as the mean diameter of the dominant population. The polydispersity index (PDI) was used to assess the uniformity of the detected colloidal size distribution.

### Antioxidant activity

The antioxidant activity of the samples was assessed using the classical 2,2-diphenyl-1-picrylhydrazyl (DPPH^•^) radical-scavenging assay, as described by Ebadi et al. (2021), with slight modifications. A 2.5 mM methanolic stock solution of DPPH^•^ was diluted to 200 µM as the working solution. For the assay, 100 µL of the sample was thoroughly mixed with 100 µL of the DPPH^•^ working solution by vortexing, then transferred to a 96-well microplate. The reaction was incubated in the dark at room temperature for 1 h to ensure complete interaction between the antioxidant compounds and the DPPH^•^ radicals. Following incubation, absorbance was measured at 515 nm using a spectrophotometer. The radical-scavenging activity was expressed as the percentage of DPPH^•^ inhibition, calculated using the following equation:

2 $$ Inhibition \% = \left( {\frac{{A_{0} - A_{s} }}{{A_{0} }}} \right) \times 100 $$

where *A*_*0*_​ is the absorbance of the control (without sample), and *A*_*s*_ is the absorbance in the presence of the sample.

### pH and titratable acidity

The pH of dessert samples was measured on days 1, 11, and 21 of refrigerated storage (4 °C) using a calibrated digital pH meter (METTLER TOLEDO, SD50, USA) at 25 °C. Prior to measurement, 10 g of the sample was homogenized with 10 mL of distilled water to ensure uniformity. For titratable acidity, 9 g of the sample was diluted with 10 mL of distilled water and titrated with 0.1 N NaOH under continuous stirring until a persistent pale-pink endpoint (30 s) was observed using phenolphthalein indicator (Latifi et al., 2021). Titratable acidity was expressed as percent lactic acid according to the following equation:

3 $$ Acidity\left( \% \right) = \left( {\frac{V \times N \times 0.09}{W}} \right) \times 100 $$

where *V* is the volume of NaOH used (mL), *N* is the normality of NaOH, *W* is the sample weight (g), and 0.09 is the milliequivalent factor of lactic acid.

### Moisture content

Moisture content was determined according to the method of Abdolmaleki et al. (2025a, b). Empty moisture dishes were dried at 105 ± 2 °C in a forced-air oven for 30 min, cooled in a desiccator to room temperature, and weighed using an analytical balance (± 0.0001 g accuracy). Approximately 2.00 g of the homogenized dessert sample was accurately weighed into the pre-dried dish, and the combined mass was recorded. Samples were dried at 105 ± 2 °C until a constant weight was reached. After each drying cycle, samples were cooled in a desiccator and reweighed. Drying continued until two consecutive measurements differed by less than 0.5 mg. Moisture content was calculated based on mass loss during drying using the following equation.

4 $$ Moisture\,(\% )\, = \left( {\frac{{W_{i} - W_{d} }}{{W_{i} }}} \right) \times 100 $$

where *W*_*1*_ is the initial sample weight (g), *W*_*d*_ is the dried sample weight (g).

### Ash content

Ash content was determined according to Daneshniya et al. (2025), using the dry ashing technique. Porcelain crucibles were pre-cleaned and heated at 550 °C for 30 min in a muffle furnace (TMax, China) to remove residual impurities. Crucibles were cooled in a desiccator and weighed using an analytical balance. Approximately 2.00 g of the homogenized dessert sample was weighed into each crucible. Samples were first dried at 105 °C for 1 h to remove moisture and then gradually heated in the muffle furnace to 550 °C. Ashing was continued at 550 °C for 4 h until light gray or white ash was obtained. The crucibles were removed, cooled in a desiccator, and weighed. To ensure complete oxidation of residual carbon, the ash was moistened with a few drops of distilled water, dried at 105 °C for 1 h, reheated at 550 °C for 30 min, cooled, and reweighed. The ash content was measured based on the following equation:

5 $$ {\mathrm{Ash}}\left( \% \right) = \frac{{(C_{w} + A_{w} ) - C_{w} }}{{S_{w} }} \times 100 $$

where *C*_*w*_ is the crucible weight, *A*_*w*_ is the ash weight and *S*_*w*_ is the crucible and sample weight before ashing (g).

### Protein content

Crude protein content was determined by the Kjeldahl method (Maleki et al., 2025). Approximately 2.00 g of homogenized dessert sample was accurately weighed into a Kjeldahl digestion flask. A catalyst mixture containing 7 g potassium sulfate and 1 g copper sulfate was added, followed by 20 mL concentrated sulfuric acid (98%). Digestion was carried out in a Kjeldahl digestion unit (Velp Scientifica, Italy) until the solution became clear and greenish, indicating complete conversion of organic nitrogen to ammonium sulfate. After cooling, the digest was diluted with distilled water and neutralized with 40% NaOH solution before steam distillation in a Kjeldahl distillation unit. The released ammonia was captured in 2% (w/v) boric acid solution containing mixed indicator. The distillate was titrated with standardized 0.1 N HCl until the endpoint color change was observed. Nitrogen content was calculated as:

6 $$ {\text{Nitrogen }}\left( \% \right) = \frac{{\left( {Vs - Vb} \right) \times N_{HCl} \times 14.007}}{{S_{w} \times 1000}} \times 100 $$

where is the volume of HCl used for the sample (mL), ${V}_{b}$ is the volume of HCl used for the blank (mL), ${N}_{HCl}$ is the normality of HCl, and *S*_*w*_ is the sample weight in grams.

7 $$ {\text{Protein }}\left( {{\% }} \right) = {\text{Nitrogen }}\left( {{\% }} \right) \times 5.7 $$

Crude protein content was calculated using a conversion factor of 5.7, appropriate for cereal-based matrices.

### Fat content

Fat content was determined using a modified Mojonnier ether extraction method (Ether extraction method, 2023). Dessert samples were equilibrated at 40 °C in a thermostatic water bath (LabTron, UK) to ensure uniform fat distribution and gently mixed. Approximately 0.60 g of homogenized sample was accurately weighed into a Mojonnier extraction flask. Warm distilled water (50 °C) was added to adjust the volume to 10 mL, followed by 2 mL ammonia solution (25%) to release fat bound to proteins and 10 mL ethanol to prevent emulsion formation. Subsequently, 25 mL diethyl ether and 25 mL petroleum ether (boiling range 40–60 °C) were sequentially added. After each addition, the flask was tightly stoppered and shaken gently for 1 min. Phase separation was facilitated by centrifugation at 3000 × g for 5 min using a laboratory centrifuge. The upper organic phase was carefully decanted into a pre-weighed extraction vessel. The extraction procedure was repeated once to ensure complete fat recovery. Combined solvent extracts were evaporated using a rotary evaporator (Across International, Australia) at 40 °C. The residue was dried in an oven at 105 °C for 30 min, cooled in a desiccator, and weighed to constant mass. The fat content was determined based on the following equation:

8 $$ {\text{Fat }}\left( {{\% }} \right) = \frac{{\left( {W_{2} - W_{1} } \right)}}{{W_{S} }} \times 100 $$

where ${W}_{1}$ is the mass of the empty extraction vessel (g), ${W}_{2}$ is the mass of the vessel plus the extracted fat after solvent evaporation (g), and ${W}_{s}$ is the mass of the sample used (g).

### Texture profile analysis

Texture profile analysis (TPA) was carried out using a texture analyzer (Saicheng Technology, TA-3000, China) equipped with a 5 kg load cell (Rahman et al., 2021). Before analysis, dessert samples were removed from refrigeration and allowed to equilibrate at 25 ± 1 °C for 1 h to ensure uniform temperature. Samples were cut into uniform cubes (20 × 20 × 20 mm) using a stainless-steel cutter to maintain consistent size and geometry. Each sample was subjected to a two-cycle compression test to 50% of its original height (10 mm deformation) using a P/100 cylindrical probe. The operating conditions were as follows; pre-test speed 1.0 mm/s, test speed 1.0 mm/s, post-test speed 1.0 mm/s, trigger force 5 g, and a 5 s interval between the two compression cycles. Force–time curves were recorded and analyzed using the instrument software to determine the following textural parameters: hardness (maximum force during the first compression, g), adhesiveness (negative work required to withdraw the probe from the sample), cohesiveness (ratio of the work during the second compression to that during the first compression), elasticity (degree of structural recovery between the two compressions), gumminess (hardness × cohesiveness), and chewiness (gumminess × elasticity). All measurements were performed in triplicate.

### Microbiological analyses

Microbiological analyses were performed to evaluate presumptive LAB counts and microbial stability during storage (Abdolmaleki et al., 2025a, b; Yoon et al., 2023). For preparation of the initial suspension, 10 g of dessert sample was aseptically weighed and homogenized with 90 mL sterile Ringer solution using a laboratory homogenizer (LabBox, RSH-1, Spain) for 2 min to obtain a uniform 10⁻^1^ dilution. Yeasts and molds were enumerated using the pour plate technique. Appropriate decimal dilutions were prepared in sterile Ringer solution, and 1 mL aliquots were transferred into sterile Petri dishes, followed by the addition of 12–15 mL of molten yeast-glucose-chloramphenicol agar. Plates were incubated inverted at 25 ± 1 °C for 5 days, and results were expressed as log CFU/g. Presumptive LAB counts were determined on MRS agar using the spread plate method. Serial dilutions were prepared, and 100 µL aliquots were spread onto MRS agar plates. Plates were incubated anaerobically at 37 °C for 72 h using an anaerobic jar system. Colonies were counted and expressed as log CFU/g. Presumptive LAB counts and mold and yeast counts were determined on days 1, 11, and 21 during refrigerated storage at 4 °C. All analyses were performed in triplicate, and the detection limit of the method was 10^2^ CFU/g. Since MRS agar is not selective or strain-specific for *Lactiplantibacillus plantarum*, and molecular or strain-specific phenotypic confirmation of the recovered colonies was not performed, the results are reported as presumptive LAB counts. Although *L. plantarum* was the only lactic acid bacterium intentionally added to F_1_–F_3_, the identity of colonies recovered after storage was not independently confirmed.

### Sensory evaluation

Sensory evaluation was conducted on day 1, day 11, and day 21 of storage to assess the organoleptic properties of the formulated desserts. A panel of 28 semi-trained evaluators (aged 22–40 years) was recruited from the Department of Food Science at QIAU (Qazvin, Iran). The participants volunteered, provided informed written or oral consent, and were aware of their right to withdraw from the study at any point before data analysis. Panelists were screened for normal sensory acuity and for the absence of food allergies and received prior training to standardize their understanding of the evaluation criteria. Samples were coded with random three-digit numbers and served monadically in a randomized complete block design under controlled sensory laboratory conditions. All samples were presented in identical opaque containers at 10 ± 2 °C. Panelists evaluated color, flavor and odor, sweetness, firmness (texture/mouthfeel), and overall acceptability using a 9-point hedonic scale (1 = dislike extremely, 9 = like extremely). Panelists were instructed to rinse their mouths with water between samples and wait at least 2 min to minimize carry-over effects. In accordance with the requirements of the Iranian National Standard (INSO No. 8437, 2018), the participants were provided with a consent form, a privacy notice regarding data protection, and the purpose of the study (Meilgaard et al., 1999). Appropriate protocols for protecting the rights and privacy of all participants were utilized during the execution of the research, indicating no coercion to participate, full disclosure of study requirements and risks, written consent of participants, no release of participant data without their knowledge, and the ability to withdraw from the study at any time. The study protocol was conducted in accordance with institutional ethical guidelines (Approval No. J13404-218).

## Statistical analysis

All experimental results were expressed as mean ± standard deviation (*n*=3). Statistical analyses were performed using XLSTAT (version 2022, Addinsoft, France). Differences among treatments were evaluated by one-way analysis of variance (ANOVA), followed by Tukey’s post-hoc test at a significance level of *p* < 0.05. Principal Component Analysis (PCA) was applied to sensory and texture profile data to explore multivariate relationships and sample discrimination. Prior to PCA, variables were standardized to a zero mean and unit variance (*Z*-score normalization) to ensure equal weighting irrespective of measurement units. The PCA model was constructed using the Pearson correlation matrix in XLSTAT. Components with eigenvalues greater than 1.0 were retained according to Kaiser’s criterion. Variables with loading values ≥|0.5| were considered to contribute significantly to component interpretation. Score and loading plots were generated to visualize clustering patterns and variable contributions. Other Graphical presentations were prepared using the Tidyplots package (Engler, 2025) in RStudio (Ver. 2026.1.0.392).

## Results and discussion

Because F_0_ contained neither BSG nor microencapsulated *L. plantarum*, comparisons between F_0_ and F_1_–F_3_ represent the overall effects of the combined formulations rather than the independent effect of BSG. However, F_1_–F_3_ received the same amount of microencapsulated culture and differed in BSG concentration. Therefore, concentration-dependent differences among these formulations are discussed in the following sections, in relation to BSG level within the combined system.

### Chemical compositions of Balangu Shirazi gum (BSG)

The chemical and physical characteristics of Balangu Shirazi gum (BSG) influence its functional performance; thus, their evaluation is essential for assessing its applicability in food matrices. The extracted gum exhibited low moisture content (6.23 ± 0.38%), which supports storage stability, and a relatively high ash content (7.12 ± 0.19%), indicating the presence of mineral constituents. Protein content was low (2.08 ± 0.06%), confirming that proteins represent a minor fraction. In contrast, the gum was predominantly composed of carbohydrates, with a high sugar content (64.71 ± 5.61%), consistent with its classification as a polysaccharide-rich hydrocolloid. Furthermore, BSG contained measurable levels of phenolic compounds (4.49 ± 0.06 mg GAE/g), which contributed to its moderate antioxidant activity, as evidenced by DPPH radical scavenging (21.01 ± 0.18%). Collectively, this composition supports the multifunctional role of BSG as both a structuring and bioactive ingredient. This chemical composition highlights its potential functional properties in food applications.

### Hydrodynamic diameter of the dispersed colloidal fraction

The hydrodynamic diameter of the colloidal species detected in the diluted dispersion was determined by DLS and expressed in nm. Particle size distribution was analyzed on a volume-weighted basis. The detected distribution was predominantly composed of a population with a mean hydrodynamic diameter of 46.08 nm, accounting for 80.10% of the measured particle volume. Additional populations were detected at 56.68 nm (7.2%), 68.78 nm (8.8%), 89.13 nm (3.5%), and 48.85 nm (0.4%). The dispersion exhibited a low polydispersity index (PDI = 0.12 ± 0.02), indicating a relatively narrow distribution among the colloidal species detected by DLS.

Importantly, the 46.08-nm hydrodynamic diameter should not be interpreted as the size of intact *L. plantarum* cells or complete probiotic-containing microcapsules. Rather, the DLS measurement reflects the dominant nanoscale colloidal population detected after dilution of the dispersion. The chemical or structural identity of this population was not independently determined. Because direct microscopic characterization by SEM or TEM was not performed, the morphology and actual dimensions of the intact probiotic-containing microcapsules could not be established in the present study. The encapsulation efficiency, determined by viable-cell recovery after dissolution of the alginate matrix, was approximately 83.12%.

### pH and acidity

**Fig. 1:**
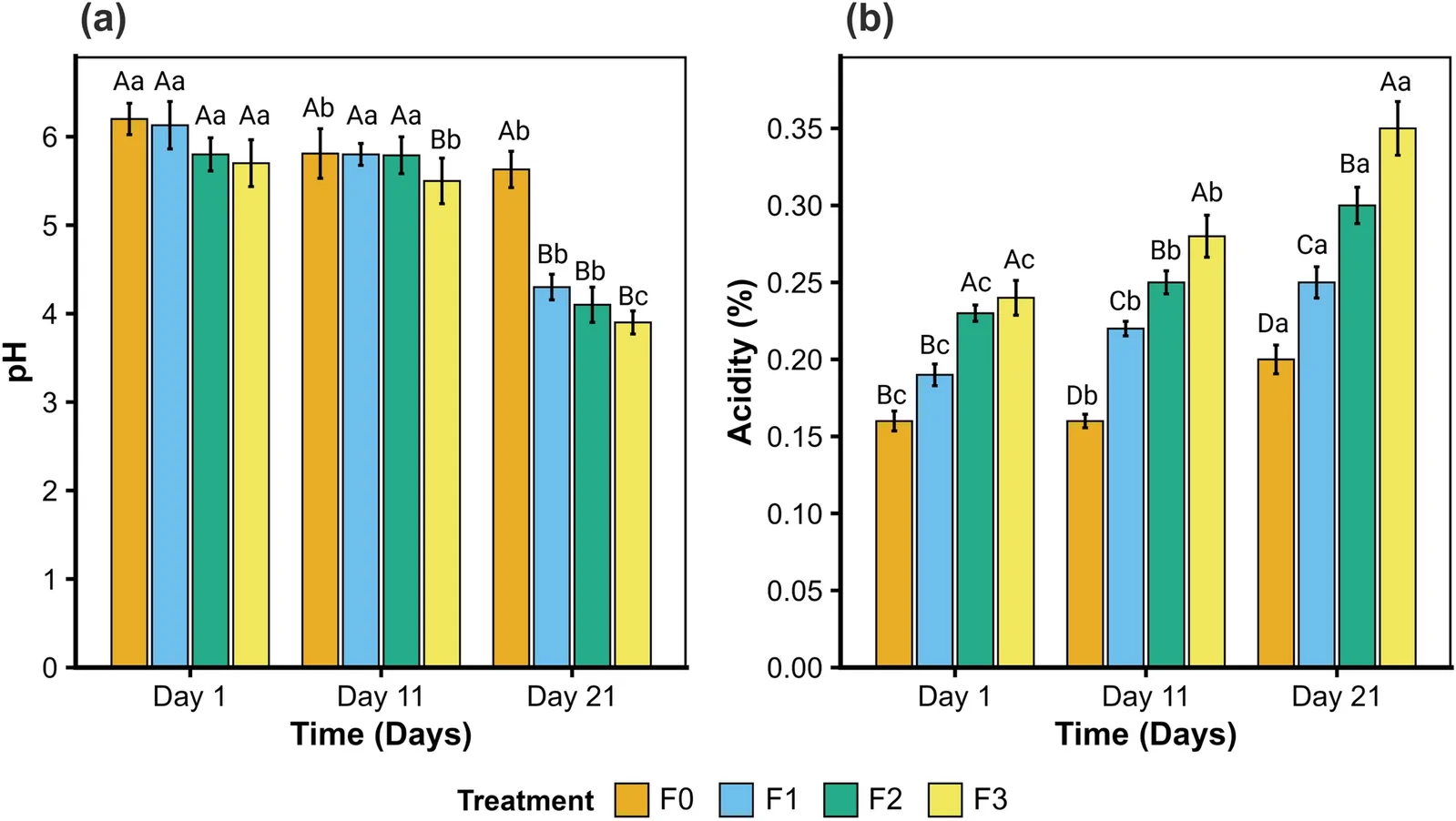
Changes in pH **(a)** and acidity **(b)** of oat-based cereal desserts during storage as affected by Balangu Shirazi (*Lallemantia royleana*) gel (BSG) levels. Values represent mean (*n*=3) ± SD (F_0_: without BSG and without microencapsulated *Lactiplantibacillus plantarum*; F_1_: 0.60 g BSG; F_2_: 1.25 g BSG; F_3_: 2.50 g BSG). Different lowercase and uppercase letters indicate significant differences among treatments over the storage period and at the same storage interval, respectively, at the *p* < 0.05 significance level

In the oat milk–based cereal dessert system, the evolution of pH and titratable acidity during storage differed significantly among the formulations. Among F_1_–F_3_, which contained a constant amount of microencapsulated culture, the extent of acidification generally increased with BSG concentration (Fig. 1), indicating a strong formulation-dependent effect on post-processing acidification. Although all treatments exhibited a gradual decline in pH over time, the extent of acidification increased markedly as BSG levels increased. By day 21, the control (F_0_) retained a relatively high pH (5.60 ± 0.20), whereas BSG-containing samples showed lower values (around 4.30 to 3.90). This trend was mirrored by titratable acidity, which increased from 0.16 ± 0.01% in F_0_ to 0.35 ± 0.02% in F_3_ at the end of storage, demonstrating a clear dose-dependent response.

In cereal-based systems, such progressive acidification during storage is primarily associated with the metabolic activity of lactic acid bacteria, which convert available carbohydrates into organic acids (Russo et al., 2017). In the present study, the greater acidification observed in the formulations containing the added microencapsulated culture may therefore be associated with acid-producing microbial activity; however, because colonies recovered on MRS agar were not confirmed using species- or strain-specific methods, the contribution of the inoculated *Lactiplantibacillus plantarum* cannot be established solely from the plate-count results. In this context, the pronounced acidification (*p* < 0.05) observed in BSG-containing samples suggests that the gum not only served as a structural agent but also may have influenced the physicochemical environment associated with continued microbial activity during storage (Ghods Rohani et al., 2020). This effect can be rationalized by considering the physicochemical properties of BSG. Its high carbohydrate content and hydrocolloid nature may enhance water retention and matrix viscosity, thereby improving the stability of the dispersed phase and limiting cellular stress (Elderwy et al., 2025). Such conditions may favor the survival and metabolic persistence of lactic acid bacteria in semi-solid systems. Although previous studies, such as those on lactic acid fermentation in calcium alginate (Wang et al., 1995) and cheese microstructure (Silva et al., 2013), highlight the point that higher gel density and cell loading increase mass-transfer resistance, as matrix density controls solute diffusion via porosity–tortuosity relationships, with denser networks impeding rather than facilitating transport, the present results suggest that differences in matrix structure associated with BSG concentration may have influenced the conditions governing acidification. However, mass transfer and substrate diffusion were not directly evaluated. Consequently, higher BSG concentrations (F_2_ and F_3_) exhibited greater reductions in pH and higher acidity, indicating greater post-acidification within these formulations rather than confirmed enhancement of *L. plantarum*-specific activity. Furthermore, the presence of phenolic compounds and their associated antioxidant activity could potentially influence microbial metabolism. However, their utilization and metabolic effects were not directly investigated in the present study (Filannino et al., 2015). The fact that these differences became more pronounced at later stages suggests formulation-dependent differences in the progression of post-acidification. Generally, the results indicate that BSG concentration was associated with differences in the extent and progression of acidification within the combined formulations. However, because MRS enumeration was not species-specific, the observed acidification cannot be attributed specifically to confirmed *L. plantarum* populations. This highlights the importance of hydrocolloid–microbe interactions in determining the stability and biochemical evolution of cereal-based functional foods.

### Moisture content

**Fig. 2:**
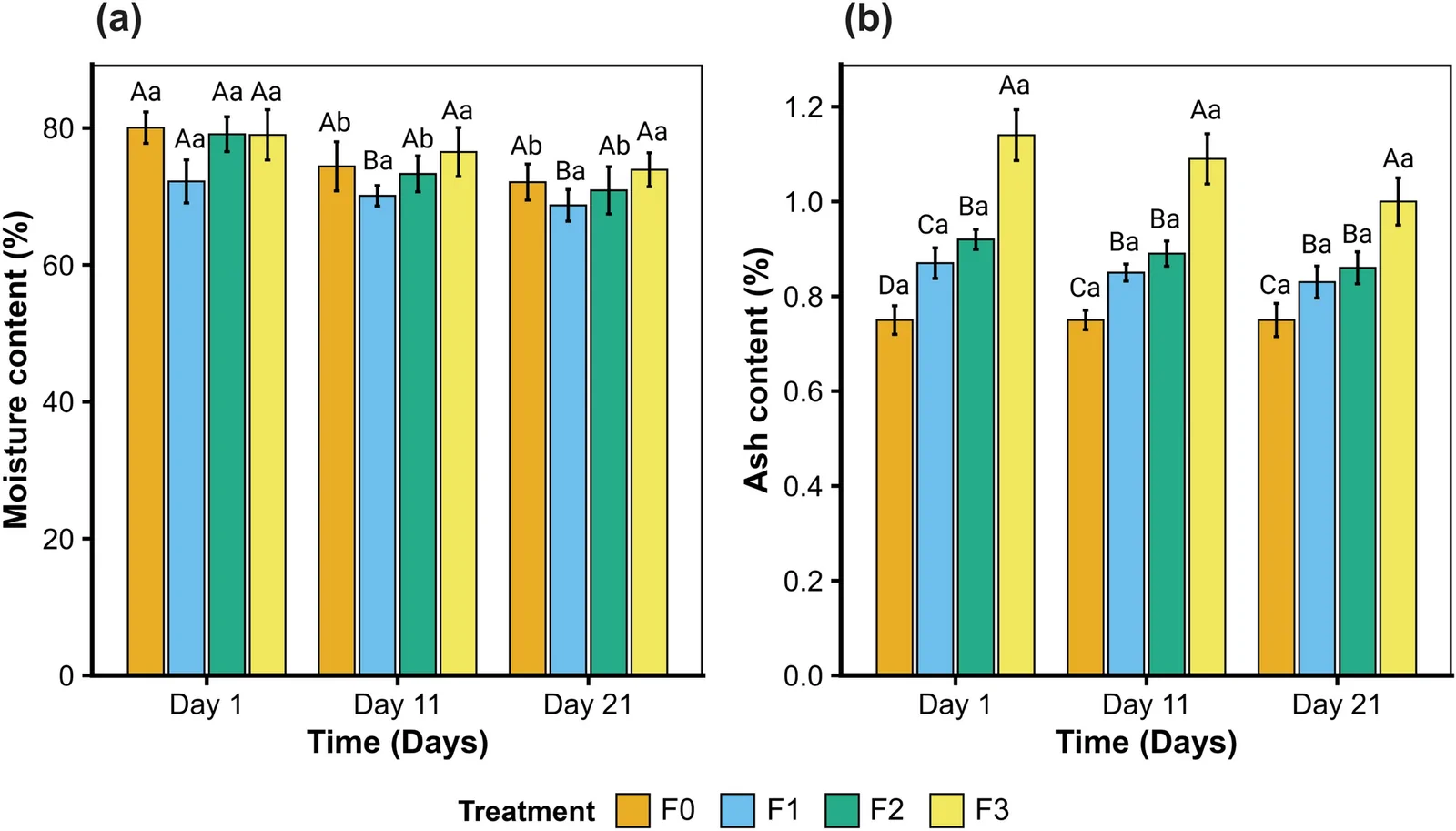
Changes in moisture **(a)** and ash **(b)** of oat-based cereal desserts during storage as affected by Balangu Shirazi (*Lallemantia royleana*) gel (BSG) levels. Values represent mean (*n*=3) ± SD of triplicate (F_0_: without BSG and without microencapsulated *Lactiplantibacillus plantarum*; F_1_: 0.60 g BSG; F_2_: 1.25 g BSG; F_3_: 2.50 g BSG). Different lowercase and uppercase letters indicate significant differences among treatments over the storage period and at the same storage interval, respectively, at the *p* < 0.05 significance level

Moisture content differed significantly according to storage time and formulation. Among F_1_–F_3_, moisture retention also depended on BSG concentration (Fig. 2a). A gradual decline was observed across all treatments during storage; however, the extent of moisture loss was clearly dependent on the level of BSG incorporation. At day 1, moisture values ranged from approximately 72% in F_1_ to 80% in F_0_, with F_2_ and F_3_ showing comparable values of 78.5 ± 2.0% and 79.0 ± 2.2%, respectively. By day 11, moisture decreased to 75.0 ± 2.3% for F_0_ and 76.0 ± 2.4% for F_3_. This trend continued at day 21, reaching 68.0 ± 1.8% (F_1_), 70.0 ± 2.0% (F_2_), and 74.0 ± 2.2% (F_3_). Notably, higher BSG levels (F_2_ and F_3_) consistently retained more moisture throughout storage, particularly at later stages. This behavior reflects the strong water-binding capacity of BSG, attributable to its polysaccharide-rich composition, which facilitates the formation of a hydrated network that immobilizes water within the matrix. Such hydrocolloid systems are known to reduce water mobility and limit moisture migration (Seth et al., 2018).

The greater moisture retention observed at higher BSG concentrations may have influenced the local environment surrounding the encapsulated cells. However, hydration around bacterial cells and its direct relationship with bacterial survival were not measured in the present study (Osojnik Črnivec et al., 2021), which may, nonetheless, support their survival. The concurrent acidification (Fig. 1) indicates that greater moisture retention occurred alongside acidification. However, the effects of water distribution on bacterial metabolism were not directly evaluated. Overall, in LAB-containing formulations, higher BSG concentrations were associated with improved moisture retention during storage.

### Ash content

Ash content increased significantly across the combined formulations as BSG concentration increased and showed a slight decline during storage (Fig. 2b). On day 1, ash values ranged from around 0.75 to 1.15% in F_3_, with intermediate values of 0.87 ± 0.04% (F_1_) and 0.92 ± 0.03% (F_2_), demonstrating a clear concentration-dependent contribution of BSG. This trend remained consistent throughout storage, although a gradual reduction was observed. By day 21, ash content decreased slightly to 0.75 ± 0.03% (F_0_) and 0.98 ± 0.05% (F_3_). The higher ash content in BSG-containing samples is directly attributable to the mineral composition of the gum itself (Sect. 3.1), confirming its contribution as a mineral-rich ingredient in the formulation (Eze et al., 2021). The slight reduction over time may be associated with structural and biochemical changes within the matrix during storage. However, in systems involving *L. plantarum*, organic acid production can promote mineral solubilization and interactions with other components, potentially affecting their distribution and the measurable ash fraction (Bahaciu et al., 2018). Moreover, the hydrocolloid network formed by BSG may influence mineral retention by stabilizing the matrix and limiting phase separation.

### Protein content

**Fig. 3:**
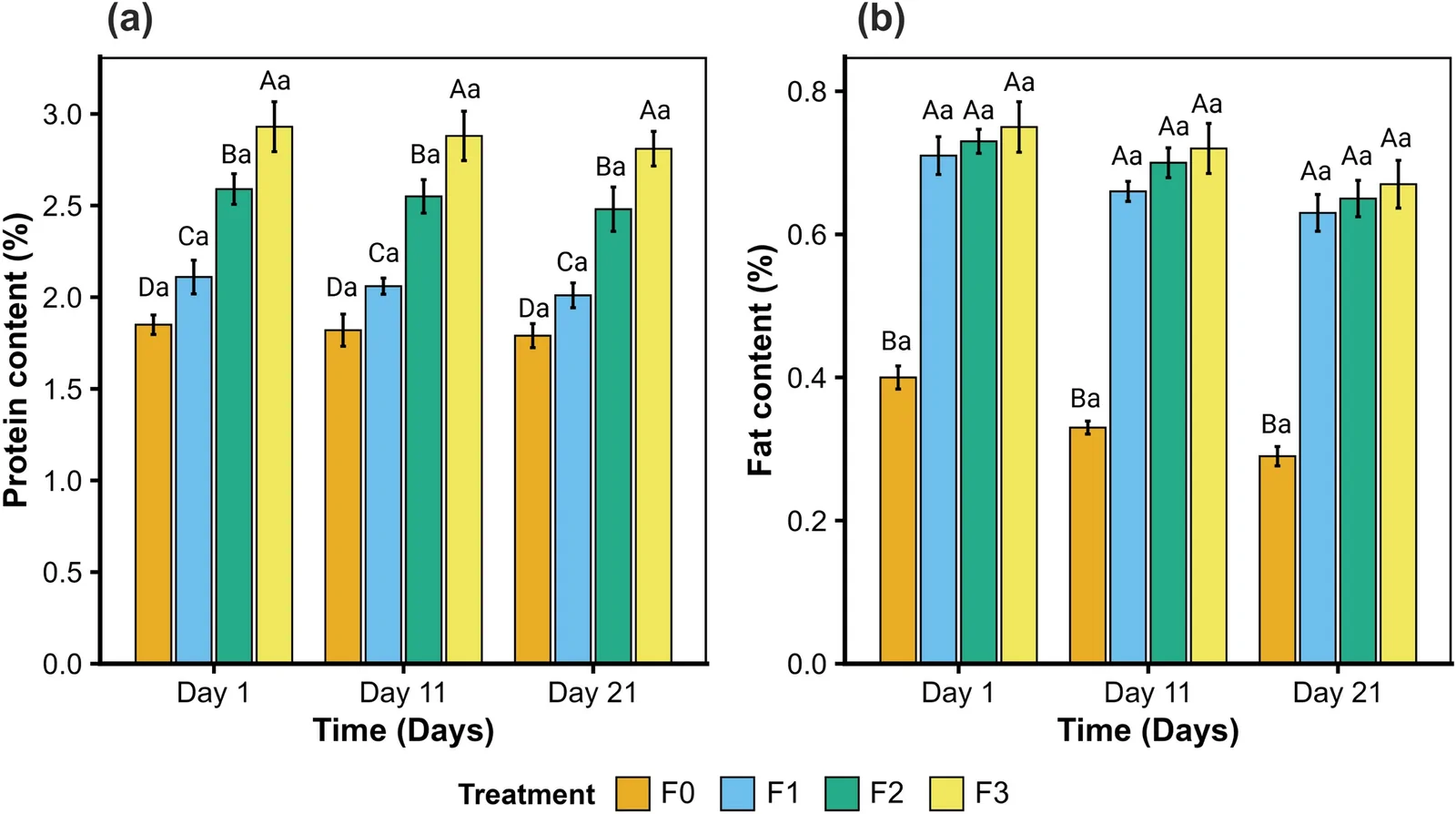
Protein content **(a)** and fat content **(b)** of oat-based cereal desserts during storage as affected by Balangu Shirazi (*Lallemantia royleana*) gel (BSG) levels. Values represent mean (*n*=3) ± SD (F_0_: without BSG and without microencapsulated *Lactiplantibacillus plantarum*; F_1_: 0.60 g BSG; F_2_: 1.25 g BSG; F_3_: 2.50 g BSG). Different lowercase and uppercase letters indicate significant differences among treatments over the storage period and at the same storage interval, respectively, at the *p* < 0.05 significance level

As shown in Fig. 3a, protein content differed significantly among formulations and increased progressively with BSG concentration across F_1_–F_3_, while only minor changes occurred during storage. On day 1, protein values increased progressively with BSG level, ranging from 1.85 ± 0.08% in F_0_ to 2.95 ± 0.12% in F_3_, with intermediate values of 2.10 ± 0.08% (F_1_) and 2.60 ± 0.11% (F_2_). This concentration-dependent trend remained consistent throughout storage. By day 11, protein content was approximately 1.82 ± 0.09% (F_0_), 2.05 ± 0.06% (F_1_), 2.55 ± 0.10% (F_2_), and 2.90 ± 0.12% (F_3_), while at day 21, values slightly decreased to 1.80 ± 0.08%, 2.00 ± 0.09%, 2.50 ± 0.11%, and 2.85 ± 0.10% for F_0_, F_1_, F_2_, and F_3_, respectively.

The higher protein content observed in BSG-containing treatments can be attributed to the gum's intrinsic protein fraction, which directly contributes to the overall composition of the dessert matrix. The slight decrease in protein during storage, previously reported (Brodziak et al., 2020), may be due to protein interactions or structural rearrangements within the system, rather than actual protein loss. Additionally, the hydrocolloid network formed by BSG may influence protein distribution and retention by stabilizing the matrix and limiting phase separation (Arab et al., 2023).

### Fat content

As shown in Fig. 3b, fat content varied across formulations and storage times, with the combined BSG–probiotic formulations exhibiting higher measured values than F_0_. On day 1, fat content increased from 0.40 ± 0.03% in F_0_ to 0.75 ± 0.05% in F_3_, with intermediate values of 0.70 ± 0.04% (F_1_) and 0.72 ± 0.03% (F_2_). During storage, a gradual decline was observed in all treatments. By day 11, fat content decreased to around 0.33% for F_0_ and 0.72% for F_3,_ and further declined at day 21 to 0.67 ± 0.04% for F_3_. Fat content remained relatively stable during storage across all treatments, consistent with the general resistance of lipid composition to change in fermented products under refrigeration (Hanuš et al., 2024). The slightly higher measured fat values in the combined formulations may reflect differences in emulsion stability and phase separation. However, the respective contributions of BSG and probiotic incorporation could not be distinguished by the present design. Minor fluctuations observed at later time points, although not statistically significant, may reflect the gradual structural rearrangement of the gel network. Additionally, *L. plantarum* has been reported to enhance oxidative stability in fermented dairy systems through its antioxidant activity (Hashemi et al., 2016), thereby preserving lipid quality during storage.

### Microbial dynamics

**Fig. 4:**
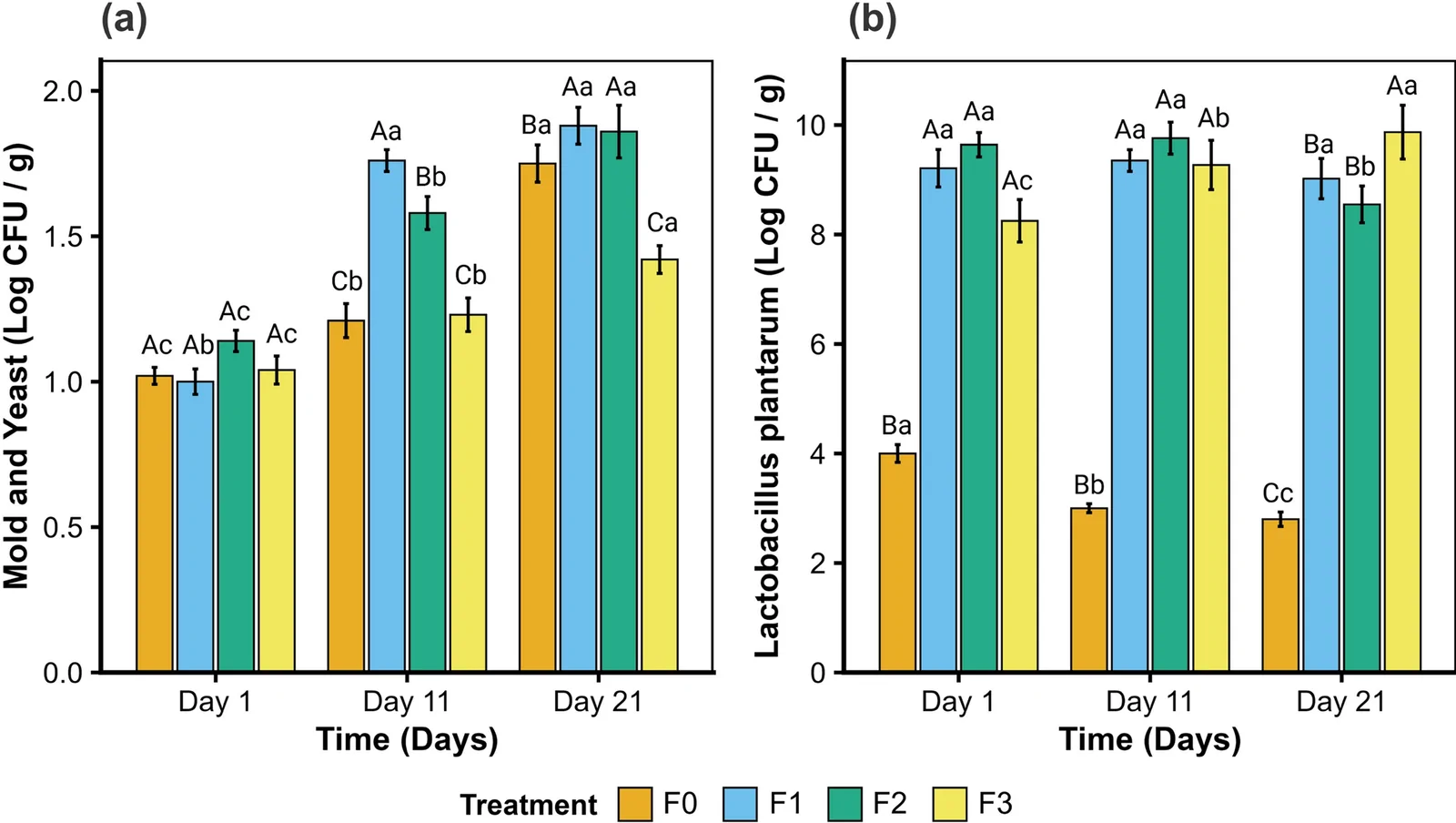
Mold and yeast counts **(a)** and *Lactobacillus plantarum* viability **(b)** of oat-based cereal desserts during storage as affected by Balangu Shirazi (*Lallemantia royleana*) gel (BSG) levels. Values represent mean (*n*=3) ± SD (F_0_: without BSG and without microencapsulated *Lactiplantibacillus plantarum*; F_1_: 0.60 g BSG; F_2_: 1.25 g BSG; F_3_: 2.50 g BSG). Different lowercase and uppercase letters indicate significant differences among treatments over the storage period and at the same storage interval, respectively, at the *p* < 0.05 significance level

The evolution of microbial populations during storage differed markedly among the formulations and, within F_1_–F_3_, according to BSG concentration (Fig. 4). Mold and yeast counts (Fig. 4a) increased progressively over time across all treatments; however, the extent of growth varied by formulation. On day 1, counts were relatively low and similar across treatments, ranging from approximately 1.00 to 1.15 log CFU/g. By day 11, microbial growth became more pronounced, with values increasing to approximately 1.20 ± 0.08 (F_0_), 1.75 ± 0.10 (F_1_), 1.60 ± 0.09 (F_2_), and 1.22 ± 0.05 log CFU/g (F_3_). On day 21, the highest counts were observed in F_1_ and F_2_, reaching 1.90 ± 0.08 and 1.85 ± 0.10 log CFU/g, respectively, compared with 1.75 ± 0.10 log CFU/g in F_0_, while F_3_ exhibited a comparatively lower value of 1.45 ± 0.08 log CFU/g.

In contrast, presumptive LAB counts recovered on MRS agar remained high in the formulations containing both BSG and microencapsulated *L. plantarum* throughout storage (Fig. 4b). On day 1, presumptive LAB counts were approximately 4.00 ± 0.20 log CFU/g in F_0_, compared with significantly higher values of 9.20 ± 0.30, 9.60 ± 0.25, and 8.20 ± 0.35 log CFU/g in F_1_, F_2_, and F_3_, respectively. During storage, counts in F0 progressively decreased to 3.00 ± 0.15 log CFU/g on day 11 and 2.80 ± 0.12 log CFU/g on day 21. Because F_0_ was not inoculated with microencapsulated *L. plantarum*, these counts represent background MRS-recoverable populations and should not be interpreted as survival of the added culture.

The formulations containing both BSG and microencapsulated cells maintained high presumptive LAB counts during storage, with F_1_ remaining at approximately 9.30 ± 0.28 to 9.00 ± 0.25 log CFU/g, F2 showing a modest decrease from 9.80 ± 0.23 to 8.50 ± 0.29 log CFU/g, and F_3_ reaching 9.90 ± 0.32 log CFU/g by day 21. Because MRS agar is not strain-specific and the recovered colonies were not confirmed by molecular or phenotypic testing, these results cannot be interpreted as a confirmed enumeration of the inoculated *L. plantarum* strain. Nevertheless, because *L. plantarum* was the only LAB intentionally added to F_1_–F_3_, it was expected to constitute the major proportion of the MRS-grown microbial population in these formulations.

These findings demonstrate maintenance of high presumptive LAB counts in the combined BSG–alginate encapsulation systems. However, because all probiotic-containing treatments received encapsulated cells and no free-cell or encapsulated-cell-only formulation was included, the respective contributions of encapsulation, BSG, and their possible interaction cannot be determined from the present experimental design. The detection of high presumptive LAB counts in F_1_–F_3_, therefore, should not be attributed independently to BSG or encapsulation. Nevertheless, because the amount of microencapsulated culture was kept constant across F_1_–F_3_, the differences among these formulations suggest an association between BSG concentration and microbial behavior within the combined system, rather than demonstrating a direct protective effect of BSG (Melchior et al., 2020).

The reduced mold and yeast growth observed in F_3_ at the later stages of storage may be associated with its lower pH and higher acidity (Fig. 1), which could create less favorable conditions for spoilage microorganisms (Riesute et al., 2021). The water-binding capacity and viscosity-modifying properties of BSG may also have contributed to environmental conditions compatible with bacterial survival. However, cellular stress, bacterial utilization of BSG carbohydrates, and the direct relationship between these matrix properties and cell survival were not evaluated in the present study.

It may further be hypothesized that the structured matrix contributed to the spatial stabilization of the encapsulated cells, as previously proposed for other polysaccharide and polymer systems (Osojnik Črnivec et al., 2021; Truong et al., 2017). Nevertheless, matrix microstructure, diffusion behavior, capsule integrity, and bacterial release were not investigated. Accordingly, these proposed explanations should be considered hypotheses consistent with the observed results rather than experimentally demonstrated mechanisms.

Overall, the combined formulations maintained high presumptive LAB counts and showed formulation-dependent differences in mold and yeast growth during storage. However, the present findings neither establish an independent protective effect of BSG or encapsulation nor demonstrate the mechanisms responsible for the observed microbial behavior.

### Sensory properties

**Fig. 5:**
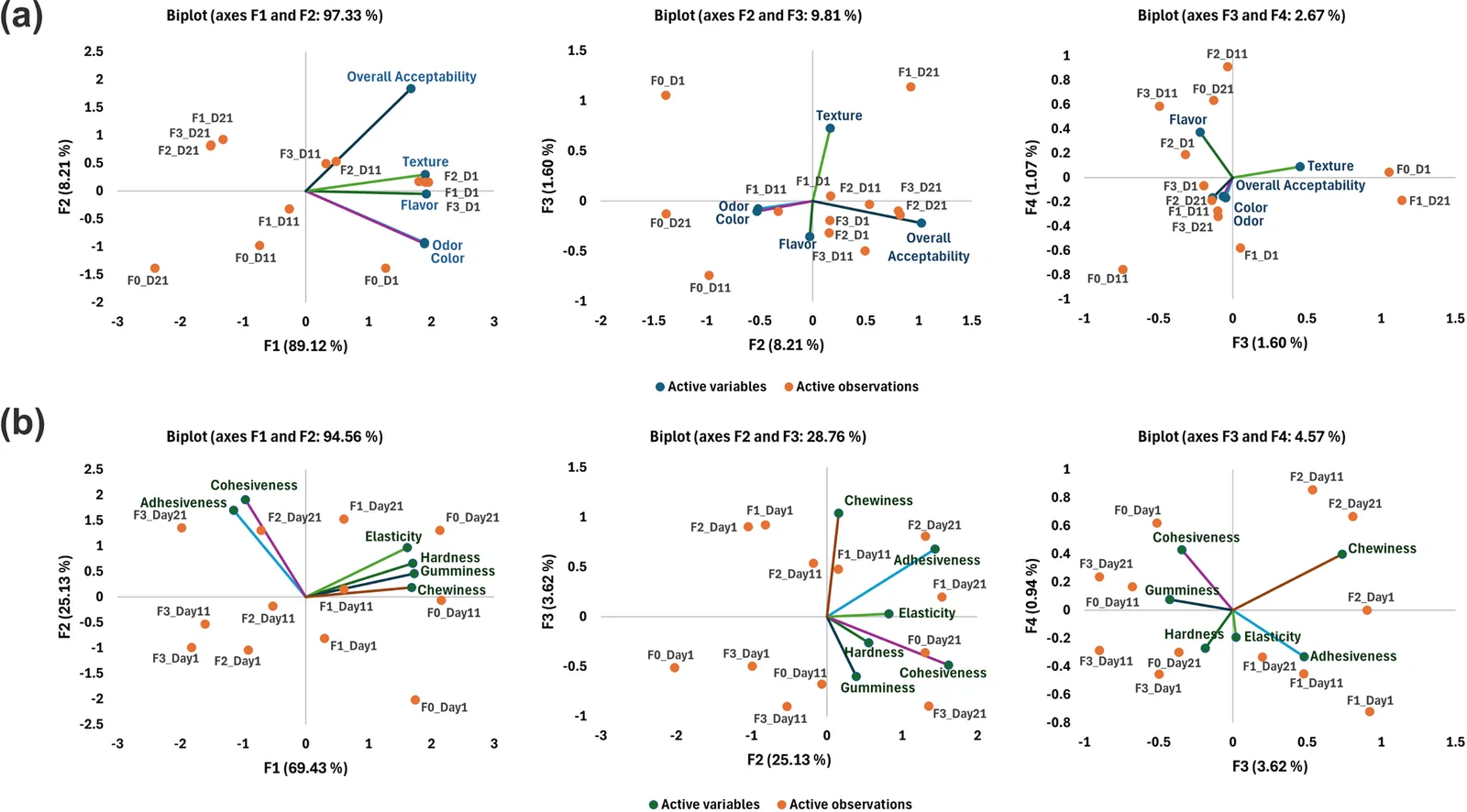
The PCA models of various sensorial **(a)** and textural **(b)** properties affected by different levels of Balangu Shirazi (*Lallemantia royleana*) gel (BSG), explored in three intervals, based on the Pearson correlation matrix, and considering up to five factors. Significant components were selected based on eigenvalues `>*1* (Kaiser’s criterion), with variables contributing meaningfully identified by loadings exceeding ±0.5. A significance level of *α*=0.05 was applied to ensure the statistical validity of the results (F_0_: without BSG and without microencapsulated *Lactiplantibacillus plantarum*; F1: 0.60 g BSG and encapsulated *L. plantarum*; F_2_: 1.25 g BSG and encapsulated *L. plantarum*; F_3_: 2.50 g BSG and encapsulated *L. plantarum*)

Principal component analysis (PCA) revealed a highly structured sensory space, with the first two components explaining 97.33% of total variance, indicating that sensory responses were governed by a limited number of latent dimensions (Fig. 5a; Tables S1–S3). A variance of 97.33% explained by the first two components in the present study is notably higher than typically reported in sensory PCA analyses, where values in the range of 70–85% are more common. This discrepancy likely reflects the nature of the variables examined, namely hedonic attributes (flavor, odor, color, texture, and overall acceptability), which are often highly intercorrelated in consumer evaluations (Ghosh & Chattopadhyay, 2012). F1 received near-equal contributions from flavor, odor, color, texture, and overall acceptability, and all attributes were well represented (cos^2^ = 0.722–0.953), identifying F1 as an integrated sensory quality axis. Positive F1 scores corresponded to superior overall sensory performance, whereas negative scores indicated global deterioration, consistent with the uniform orientation of all sensory vectors. F_2_ captured secondary variation, primarily driven by overall acceptability, with minor contributions from color and odor. Its modest representation (low cos^2^) indicates that it serves as a discriminating but non-dominant axis, separating samples based on the relative weighting of holistic acceptability versus specific descriptors. Higher-order components (F3–F4) explained minimal variance and reflected minor texture- and flavor-related nuances, supporting but not altering the primary interpretation.

A strong storage-time effect was evident across all treatments. Samples shifted progressively from positive F1 values at day 1 to negative values by day 21, indicating a coordinated decline across all sensory attributes. This consistent trajectory, as previously reported for fermented milk (Melia et al., 2024) and yogurt (Lestari et al., 2022), demonstrates that storage time was the primary driver of sensory degradation, affecting the entire sensory profile rather than isolated traits. Formulation effects were also apparent. On day 1, the combined BSG–probiotic formulations generally clustered in the higher-quality region, whereas F_0_ showed a less favorable sensory balance; this is consistent with studies reporting that the addition of hydrocolloids leads to initial improvements in sensory scores in dairy products (Garmaroudi et al., 2025; Ghods et al., 2021). By day 11, formulations F_2_ and F_3_ showed higher PC1 scores than formulation F_1_ and especially F_0_, indicating comparatively better retention of the overall sensory profile. These differences may be associated with changes in moisture retention and matrix structure; however, the independent contributions of BSG and probiotic incorporation cannot be distinguished using the present experimental design. By day 21, all samples had deteriorated; however, treated samples remained less negative than the control, confirming mitigation of sensory loss and underscoring that hydrocolloids may delay but do not prevent sensory decline (Kadağan & Arslan, 2021). Among them, F_1_ showed relatively better retention of acceptability- and texture-related characteristics, while F_2_ and F_3_ also remained clearly separated from the more degraded control.

### Textural properties

Principal component analysis (PCA) revealed a highly structured textural space, with the first two components explaining 94.56% of total variance (F_1_ = 69.43%, F_2_ = 25.13%), indicating that textural differences were governed by a limited number of latent dimensions (Fig. 5b; Tables S4–S6). F1 was primarily defined by hardness, gumminess, chewiness, and elasticity, with smaller contributions from adhesiveness and cohesiveness. These variables were well represented on F1 (cos^2^ = 0.800–0.914), indicating it as the main axis of structural firmness and mechanical resistance (Özcan, 2013), with positive scores corresponding to firmer, more elastic textures and negative scores to softer structures. F2 captured secondary variation, mainly associated with cohesiveness and adhesiveness, which were better represented on this axis. This indicates that F2 reflects internal binding and stickiness-related behavior, acting as a discriminating but non-dominant dimension that separates samples by cohesion/adhesion rather than by bulk firmness.

Higher-order components (F3-F4) explained minimal variance and reflected minor residual differences related to chewiness, adhesiveness, and cohesiveness, without altering the main interpretation. A treatment-driven effect was evident along F_1_. F_0_ samples consistently showed positive F1 scores, indicating higher hardness, gumminess, chewiness, and elasticity, whereas F_3_ samples were strongly negative, corresponding to the softest texture. Since F_1_–F_3_ had the same microcapsule loading, the observed differences among these formulations may be attributed to the BSG concentration in the combined system. However, comparisons with F_0_ cannot isolate the effect of BSG from that of probiotic fortification. F_2_ also remained negative but less extreme, while F_1_ occupied an intermediate position. This distribution indicates a progressive shift from a firm, resistant matrix toward a softer structure, with the magnitude of the effect following F_3_ > F_2_ > F_1_ relative to F_0_. A storage-time effect was also observed, primarily along F_2_. Across treatments, samples generally shifted toward higher F2 values with storage, indicating increased cohesiveness and adhesiveness over time; although this does not resemble any typical pattern (Elhamirad & Hesari, 2022; Gerçekaslan & Aktaş, 2020), it may stem from the matrix specifications with BSG. This trend was evident in F_0_, F_1_, and F_2_, and in F_3_, which, despite remaining soft (negative F_1_), transitioned toward positive F2 values at later stages. These results suggest that storage mainly influenced secondary textural organization rather than the primary firmness gradient (Coggins et al., 2010).

## Conclusion

Overall, this study demonstrates the feasibility of developing an oat milk-based functional dessert containing both microencapsulated *Lactiplantibacillus plantarum* and Balangu Shirazi gum. Compared with the untreated control, the combined formulations exhibited improved moisture retention and compositional stability, maintained high presumptive LAB counts, and showed distinct sensory and textural behavior during refrigerated storage. Because the probiotic loading was constant across F_1_–F_3_, the concentration-dependent differences observed among these treatments suggest that BSG concentration contributed to the behavior of the combined system. Higher BSG concentrations were generally associated with greater moisture retention, compositional changes, increased acidification, and softer textures.

However, because BSG-only, probiotic-only, and free-cell treatments were not included, the independent effects of BSG, probiotic incorporation, encapsulation, and their interactions could not be determined. In particular, the present study cannot determine whether probiotic incorporation independently affected texture, acidity, flavor, or sensory acceptability, or whether BSG mitigated any probiotic-induced changes in quality. Therefore, the findings should be interpreted as demonstrating the performance of the combined BSG–microencapsulated probiotic formulations rather than proving the independent functionality of BSG. Accordingly, the novelty of this study lies in the development and refrigerated-storage characterization of an oat milk-based dessert integrating BSG and microencapsulated *L. plantarum*, rather than in demonstrating that BSG counteracted changes caused by probiotic fortification. Future studies employing a factorial design should separately evaluate these components and their interactions.

## Supplementary Information

Below is the link to the electronic supplementary material.

Supplementary file1 (DOCX 32 KB)
