# Supplementary material for: Development and characterization of a functional oat milk-based cereal dessert fortified with microencapsulated Lactobacillus plantarum and Balangu Shirazi (Lallemantia royleana) gum
Source: Food Sci Biotechnol. 2026 Sep 18;35(14):4099–114. doi: 10.1007/s10068-026-02302-w (PMC13755113; doi:10.1007/s10068-026-02302-w)
Supplement: Supplementary file 1 [file 10068_2026_2302_MOESM1_ESM.docx]

**Supporting information**

**Development and characterization of a functional oat milk–based cereal dessert fortified with microencapsulated *Lactobacillus plantarum* and Balangu Shirazi (*Lallemantia royleana*) gum**

Milad Daneshniya^†,1^, Parham Joolaei Ahranjani^†,2^, Kamine Dehghan^†,2^, Farzaneh Abdolmaleki^*,1^, Giovanna Ferrentino^*,2^

^1^Department of Food Science and Technology, Qa.C., Islamic Azad University, Qazvin, Iran. ([miladdaneshniya@gmail.com](mailto:miladdaneshniya@gmail.com))

^2^Faculty of Agricultural, Environmental and Food Sciences, Free University of Bolzano, Piazza Università, 1, Bolzano, 39100, Italy. ([Parhamjoolaei@gmail.com](mailto:Parhamjoolaei@gmail.com), [Kaminehdehghan@yahoo.com](mailto:Kaminehdehghan@yahoo.com))

^†^These authors contributed equally to this work.

Corresponding authors: [giovanna.ferrentino@unibz.it](mailto:giovanna.ferrentino@unibz.it), [a.abdolmaleki@qiau.ac.ir](mailto:a.abdolmaleki@qiau.ac.ir)

**Table S1.** Contribution (%) of sensory attributes to the principal components (PCs), indicating the relative importance of each attribute in explaining variability among the samples.

|  | **F1** | **F2** | **F3** | **F4** | **F5** |
| --- | --- | --- | --- | --- | --- |
| **Flavor** | 21.394 | 0.053 | 17.276 | 61.224 | 0.053 |
| **Odor** | 20.764 | 16.460 | 0.807 | 12.230 | 49.739 |
| **Color** | 20.716 | 17.068 | 1.478 | 10.560 | 50.177 |
| **Texture** | 20.915 | 1.700 | 73.741 | 3.616 | 0.028 |
| **Overall Acceptability** | 16.212 | 64.719 | 6.697 | 12.370 | 0.002 |

**Table S2.** Squared cosine (cos²) values of sensory attributes on the principal components, representing the quality of representation of each attribute in the PCA space.

|  | **F1** | **F2** | **F3** | **F4** | **F5** |
| --- | --- | --- | --- | --- | --- |
| **Flavor** | 0.953* | 0.000 | 0.014 | 0.033 | 0.000 |
| **Odor** | 0.925* | 0.068 | 0.001 | 0.007 | 0.000 |
| **Color** | 0.923* | 0.070 | 0.001 | 0.006 | 0.000 |
| **Texture** | 0.932* | 0.007 | 0.059 | 0.002 | 0.000 |
| **Overall Acceptability** | 0.722* | 0.266 | 0.005 | 0.007 | 0.000 |

*Values correspond to each variable to the factor for which the squared cosine is the largest.

**Table S3.** Factor scores of samples on the principal components, showing the positioning of each sample in relation to the sensory attributes.

|  | **F1** | **F2** | **F3** | **F4** | **F5** |
| --- | --- | --- | --- | --- | --- |
| **F0_D1** | 1.848 | -1.108 | 0.561 | 0.022 | 0.002 |
| **F0_D11** | -1.067 | -0.783 | -0.394 | -0.363 | -0.005 |
| **F0_D21** | -3.488 | -1.107 | -0.068 | 0.305 | -0.005 |
| **F1_D1** | 2.620 | 0.135 | 0.028 | -0.278 | 0.002 |
| **F1_D11** | -0.376 | -0.260 | -0.053 | -0.132 | 0.021 |
| **F1_D21** | -1.917 | 0.741 | 0.606 | -0.090 | -0.002 |
| **F2_D1** | 2.838 | 0.124 | -0.169 | 0.091 | -0.009 |
| **F2_D11** | 0.701 | 0.427 | -0.018 | 0.437 | -0.004 |
| **F2_D21** | -2.192 | 0.661 | -0.074 | -0.089 | 0.011 |
| **F3_D1** | 2.765 | 0.128 | -0.103 | -0.032 | -0.006 |
| **F3_D11** | 0.471 | 0.396 | -0.263 | 0.282 | 0.012 |
| **F3_D21** | -2.203 | 0.645 | -0.052 | -0.153 | -0.016 |

**Table S4.** Contribution (%) of samples to the principal components (PCs) based on textural properties, indicating the influence of each sample in defining the PCA structure.

|  | **F1** | **F2** | **F3** | **F4** | **F5** | **F6** |
| --- | --- | --- | --- | --- | --- | --- |
| **Hardness** | 21.408 | 5.277 | 3.110 | 12.852 | 26.654 | 30.699 |
| **Adhesiveness** | 9.753 | 35.416 | 20.896 | 19.332 | 4.374 | 10.229 |
| **Cohesiveness** | 6.788 | 44.746 | 10.659 | 32.367 | 0.084 | 5.355 |
| **Elasticity** | 19.199 | 11.555 | 0.038 | 6.545 | 62.191 | 0.472 |
| **Gumminess** | 21.947 | 2.588 | 16.323 | 1.028 | 4.983 | 53.131 |
| **Chewiness** | 20.905 | 0.417 | 48.974 | 27.876 | 1.714 | 0.114 |

**Table S5.** Squared cosine (cos²) values of samples on the principal components derived from textural properties, reflecting the quality of representation of each sample in the PCA space.

|  | **F1** | **F2** | **F3** | **F4** | **F5** | **F6** |
| --- | --- | --- | --- | --- | --- | --- |
| **Hardness** | 0.892* | 0.080 | 0.007 | 0.007 | 0.009 | 0.005 |
| **Adhesiveness** | 0.406 | 0.534* | 0.045 | 0.011 | 0.002 | 0.002 |
| **Cohesiveness** | 0.283 | 0.675* | 0.023 | 0.018 | 0.000 | 0.001 |
| **Elasticity** | 0.800* | 0.174 | 0.000 | 0.004 | 0.022 | 0.000 |
| **Gumminess** | 0.914* | 0.039 | 0.035 | 0.001 | 0.002 | 0.009 |
| **Chewiness** | 0.871* | 0.006 | 0.106 | 0.016 | 0.001 | 0.000 |

*Values correspond to each variable to the factor for which the squared cosine is the largest.

**Table S6.** Factor scores of samples on the principal components derived from textural properties, showing the positioning of each sample in relation to the measured textural parameters.

|  | **F1** | **F2** | **F3** | **F4** | **F5** | **F6** |
| --- | --- | --- | --- | --- | --- | --- |
| **F0_Day1** | 2.489 | -2.237 | -0.350 | 0.302 | -0.142 | 0.194 |
| **F0_Day11** | 3.083 | -0.074 | -0.462 | 0.082 | 0.052 | -0.234 |
| **F0_Day21** | 3.054 | 1.447 | -0.248 | -0.146 | 0.071 | -0.057 |
| **F1_Day1** | 0.427 | -0.902 | 0.629 | -0.351 | -0.361 | -0.030 |
| **F1_Day11** | 0.866 | 0.167 | 0.327 | -0.221 | 0.266 | -0.002 |
| **F1_Day21** | 0.867 | 1.693 | 0.135 | -0.163 | -0.057 | 0.256 |
| **F2_Day1** | -1.305 | -1.158 | 0.618 | 0.000 | -0.026 | -0.161 |
| **F2_Day11** | -0.745 | -0.197 | 0.367 | 0.418 | 0.123 | -0.008 |
| **F2_Day21** | -1.019 | 1.449 | 0.552 | 0.325 | 0.072 | 0.040 |
| **F3_Day1** | -2.599 | -1.100 | -0.340 | -0.222 | 0.265 | 0.045 |
| **F3_Day11** | -2.291 | -0.588 | -0.615 | -0.139 | 0.055 | 0.045 |
| **F3_Day21** | -2.824 | 1.501 | -0.614 | 0.116 | -0.317 | -0.088 |
